# Differential immune response modulation in early *Leishmania amazonensis* infection of BALB/c and C57BL/6 macrophages based on transcriptome profiles

**DOI:** 10.1038/s41598-019-56305-1

**Published:** 2019-12-27

**Authors:** Juliana Ide Aoki, Sandra Marcia Muxel, Ricardo Andrade Zampieri, Karl Erik Müller, Audun Helge Nerland, Lucile Maria Floeter-Winter

**Affiliations:** 1 Department of Physiology, Institute of Bioscience, University of São Paulo, São Paulo, Brazil; 2 Department of Clinical Science, University of Bergen, Bergen, Norway; 3 Department of Internal Medicine, Drammen Hospital, Drammen, Norway

**Keywords:** Parasitic infection, Transcriptomics

## Abstract

The fate of *Leishmania* infection can be strongly influenced by the host genetic background. In this work, we describe gene expression modulation of the immune system based on dual global transcriptome profiles of bone marrow-derived macrophages (BMDMs) from BALB/c and C57BL/6 mice infected with *Leishmania amazonensis*. A total of 12,641 host transcripts were identified according to the alignment to the *Mus musculus* genome. Differentially expressed genes (DEGs) profiling revealed a differential modulation of the basal genetic background between the two hosts independent of *L. amazonensis* infection. In addition, in response to early *L. amazonensis* infection, 10 genes were modulated in infected BALB/c *vs*. non-infected BALB/c macrophages; and 127 genes were modulated in infected C57BL/6 *vs*. non-infected C57BL/6 macrophages. These modulated genes appeared to be related to the main immune response processes, such as recognition, antigen presentation, costimulation and proliferation. The distinct gene expression was correlated with the susceptibility and resistance to infection of each host. Furthermore, upon comparing the DEGs in BMDMs *vs*. peritoneal macrophages, we observed no differences in the gene expression patterns of *Jun*, *Fcgr1* and *Il1b*, suggesting a similar activation trends of transcription factor binding, recognition and phagocytosis, as well as the proinflammatory cytokine production in response to early *L. amazonensis* infection. Analysis of the DEG profile of the parasite revealed only one DEG among the 8,282 transcripts, indicating that parasite gene expression in early infection does not depend on the host genetic background.

## Introduction

*Leishmania* is a protozoan parasite and the causative agent of several clinical infections, generically known as leishmaniases. In general, these infections are characterized by cutaneous, mucosal or visceral manifestations^1,2^. Leishmaniases are considered neglected tropical diseases by the World Health Organization. There is no vaccine available to prevent the disease due to a range of factors, such as diversity among *Leishmania* species and the interaction of these parasites with the host immune system^3–6^. Treatment can be complicated since most of the drugs available are expensive and toxic and may require long treatment regimens^7,8^. Furthermore, resistance to several commonly used drugs has been reported^9^. In humans, *L. amazonensis* infection can cause chronic cutaneous lesions, although diffuse cutaneous and visceral manifestations have been reported^1,7^.

The immune response to *Leishmania* involves a complex range of cells. Neutrophils and monocytes are first recruited to the site of the insect bite, which leads to the differentiation of macrophages; this differentiation is followed by the recognition and phagocytosis of the parasite, as well as the induction of a range of inflammatory signals. Other phagocytes, such as dendritic cells, also play important roles since they induce the response in other inflammatory tissues. However, macrophages that play a critical roles in the establishment of infection, as they are the main host cells for *Leishmania* replication inside the phagolysosome^10–13^. The infection is characterized by Th1 cell-mediated production of interferon gamma (IFN-γ), tumor necrosis factor alpha (TNF-α) and granulocyte macrophage colony-stimulating factor (GM-CSF), which polarizes macrophages to the proinflammatory M1 phenotype and increases nitric oxide synthase 2 (NOS2) and nitric oxide (NO) levels, resulting in parasite control, or by Th2 cell-mediated production of interleukin (IL) 4 (IL4), IL13, IL10, tumor growth factor beta (TGFβ) and macrophage colony-stimulating factor (M-CSF), which polarizes macrophages to an anti-inflammatory M2 phenotype and increases arginase 1 and polyamine production, resulting in parasite replication^3,14–17^. However, the parasite is able to subvert macrophage killing mechanisms through the modification of host cytokine expression, preventing antigen display by MHC class II molecules and reducing NO production with consequent amastigote differentiation and proliferation^11,12^.

*Leishmania* infection in murine models has been extensively characterized and varies according to the parasite species and host genetic background^3,18–22^. Progressive disease occurs due to impaired cellular immunity, with dysfunction of T cells, macrophages, or both^23^. Regulation of the host immune response to *Leishmania* has been well defined in *L. major* model in which the BALB/c mouse strain is susceptible to infection due to early bursts of IL4 that lead to disease progression. On the other hand, the C57BL/6 mouse strain is resistant to infection due to a dominant Th1-type response leading to infection control^13,18–20,24^. Experimental murine infections with *L. amazonensis* have demonstrated distinct susceptibilities compared to those for *L. major*^25,26^. *L. amazonensis* induces severe lesions upon cutaneous inoculation in susceptible BALB/c mice, while the same parasite causes only moderate lesions in resistant C57BL/6 mice^21,27^. Such variations in infection have been observed as differences in the lesion size, parasite burden, cellular activation and Th1/Th2 ratio between the different infected strains^21,25,28^.

Furthermore, studies involving knockout mouse strains have revealed interesting data concerning the response of the host to *Leishmania* infection. Targeted deletion of the *Il4* and *Il10* genes results in a minimal effects on the development of *L. amazonensis*^29^ and *L. major* infections^30^, due to reduced IL12 receptor expression, which leads to reduced IL12 responsiveness and, consequently, to impairment of the Th1 response^31^. In *Tlr4*- and *MyD88*- deficient mice, *L. amazonensis* shows increased *in vitro* infectivity; in contrast *Tlr2*-deficient mice exhibited a decreased parasite loads, indicating that this receptor is required for disease progression^32^.

Based on these findings, we analyzed the modulation of the early immune responses defined by the dual transcriptome profiles of BMDMs from the BALB/c and C57BL/6 mouse strains after infection with *L. amazonensis* for 4 h. Previous transcriptomic data have revealed novel information about the coordinated response of *Leishmania*-infected macrophages^33–36^ and about parasite biology, physiology and gene expression modulation^37–42^. In this work, we identified a total of 12,641 total mouse transcripts, and analyses of the DEGs profile involved in immune response modulation confirmed the existence of differences between these two hosts that can regulate susceptibility and resistance to *L. amazonensis* infection. Interestingly, the parasite transcriptome profile showed only one DEG, a noncoding RNA, indicating that the parasite presents no modulation of gene expression in early infection regardless of the host genetic background.

## Results

### BMDMs from BALB/c mice exhibited a lower infection index than those from C57BL/6 mice at 4 h after infection

BMDMs from the BALB/c and C57BL/6 mouse strains were infected with *L. amazonensis* (MOI 5:1), and the infection index was analyzed at 4 h after infection. First, no significant differences were observed in the infection rate or the number of intracellular parasites per infected macrophage (Fig. S1A,B). However, the infection index was significantly lower in infected BALB/c than in infected C57BL/6 macrophages (Fig. S1C).

### Host transcriptome profiling revealed greater gene expression modulation in BMDMs from C57BL/6 mice than in BALB/c mice in response to *L. amazonensis* infection

Transcriptomic analyses were performed on five independent biological replicates per analysis of BMDMs from BALB/c and C57BL/6 mice infected or not infected with *L. amazonensis* for 4 h, using Illumina NovaSeq. 6000 sequencing, which generated millions of reads. The sequencing data are available in the NCBI BioProject database (https://www.ncbi.nlm.nih.gov/bioproject/) under accession numbers PRJNA481041 and PRJNA481042 and in the Sequence Read Archive (SRA) database (https://www.ncbi.nlm.nih.gov/sra) under accession numbers SRP156183 and SRP156466. The RNA-seq data were aligned to the *M. musculus* reference genome, and 12,641 transcripts were identified (Table S1).

**Figure 1:**
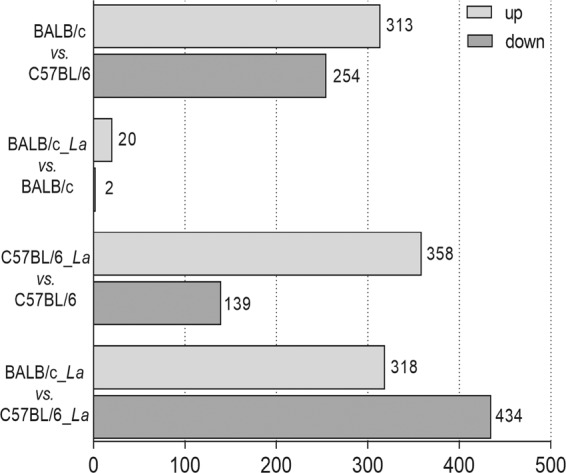
Transcriptome profiles of BMDMs from BALB/c and C57BL/6 mice infected with *L. amazonensis*. Differential gene expression profiles of BMDMs from BALB/c and C57BL/6 mice infected with *L. amazone*nsis, presented as the numbers of upregulated (light gray) and downregulated (dark gray) transcripts in the following comparisons: non-infected BALB/c *vs*. non-infected C57BL/6 macrophages; infected BALB/c *vs*. non-infected BALB/c macrophages; infected C57BL/6 *vs*. non-infected C57BL/6 macrophages; and infected BALB/c *vs*. infected C57BL/6 macrophages. The data are from five independent biological replicates, considering a fold change ≥ 2 and a *p*-value < 0.05. La, *L. amazonensis*.

Analysis of DEGs with a statistical significance threshold of a fold change ≥ 2 and a *p*-value < 0.05 revealed differential basal backgrounds in non-infected BALB/c *vs*. non-infected C57BL/6 macrophages; specifically, 313 genes were upregulated, and 254 genes were downregulated. Comparison of BALB/c_*La vs*. BALB/c macrophages revealed only 20 upregulated genes and 2 downregulated genes. In contrast, comparison of C57BL/6_*La vs*. C57BL/6 macrophages revealed 358 upregulated genes and 139 downregulated genes, and comparison of BALB/c_*La vs*. C57BL/6_*La* macrophages revealed 318 upregulated genes and 434 downregulated genes (Fig. 1).

In addition, we generated volcano plots comparing the fold changes in expression (log_2_) with the corresponding adjusted *p*-values (-log_10_) (Fig. S2A) and volume plots comparing the fold changes in expression (log_2_) with the volumes (Fig. S2B). Based on these results, we identified the five most highly modulated transcripts among the comparisons (Table S2). Functional annotation and gene enrichment analyses were performed using the Gene Ontology (GO) and Kyoto Encyclopedia of Genes and Genomes (KEGG) databases. KEGG enrichment analysis showed the 20 most differentially regulated pathways among the samples (Fig. S2C).

**Figure 2:**
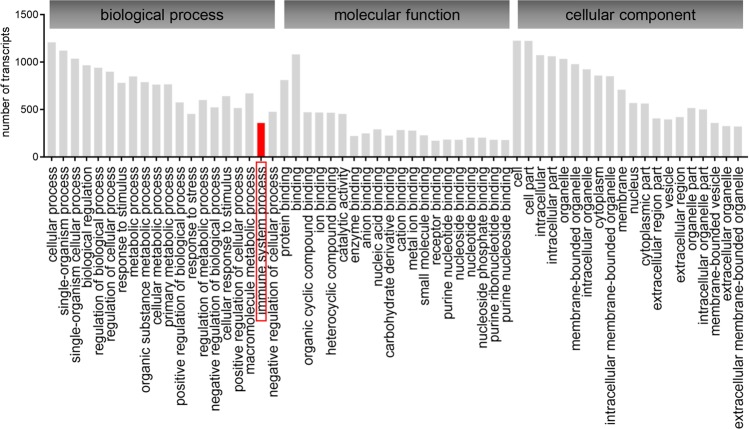
GO enrichment analysis of DEGs in BMDMs from BALB/c and C57BL/6 mice in response to *L. amazonensis* infection. The GO enrichment analysis results are presented as numbers of transcripts distributed in three main categories: biological process, molecular function and cellular component. Immune system processes were the focus of this work.

RNA-seq generates a large amount of information that can be analyzed from various perspectives. According to GO enrichment analysis of the DEGs, the most modulated subcategories were associated with biological processes, molecular functions and cellular components (Fig. 2). In this work, we focused on the immune system process term, comprising 361 modulated transcripts (Table S3), to elucidate how the host genetic background differences can define the fate of *L. amazonensis* infection.

### The gene expression modulation patterns revealed higher immune response activation in BMDMs from C57BL/6 mice than in BALB/c mice in response to *L. amazonensis* infection

Among the 361 modulated transcripts related to immune system processes, 150 of them appeared to differ in expression in non-infected BALB/c *vs*. C57BL/6 macrophages, indicating the existence of differential basal gene expression in these two host backgrounds, independent of *L. amazonensis* infection. After *L. amazonensis* infection, we observed only 10 modulated genes in BALB/c_*La vs*. BALB/c macrophages; 127 modulated genes in C57BL/6_*La vs*. C57BL/6 macrophages; and 221 modulated genes in C57BL/6_*La vs*. BALB/c_*La* macrophages (Table 1).

**Table 1:** Profile of DEGs involved in immune system processes in BMDMs from BALB/c and C57BL/6 in response to *L. amazonensis* infection.

comparison	downregulated genes	upregulated genes	*p*-value	FDR
BALB/c *vs*. C57BL/6	*Adgre1*, *AF251705*, *Ang*, *Apobec3*, *Asb2*, *Batf3*, *Blnk*, *Bst2*, *C1qa*, *C1qb*, *C1qc*, *C5ar2*, *Camp*, *Ccl5*, *Ccr2*, *Cd300a*, *Cd4*, *Cd40*, *Cd79b*, *Clec1b*, *Clec2d*, Clec4a2, *Ctsh*, *Cxcl10*, *Cxcl9*, *Emr1*, *Erbb2ip*, *Fcgr4*, *Fcna*, *Gbp2*, *Gbp3*, *Gbp5*, *Gbp7*, *Hfe*, *Ifit1bl1*, *Iigp1*, *Il15*, *Il18bp*, *Irf1*, *Irgm2*, *Itga4*, *Itgad*, *Itgal*, *Lcn2*, *Lgals1*, *Ly86*, *Marco*, *Mertk*, *Mill2*, *Pde4b*, *Pnp*, *Prdm1*, *S100a8*, *Samhd1*, *Skil*, *Slamf7*, *Slc11a1*, *Slc40a1*, *Smad6*, *Tfrc*, *Tgtp1*, *Tlr8*, *Tmem176a*, *Tmem176b*, *Trim34a*, *Vcam1*, *Vegfa*, *Vsig4*, *Wwp1*	*Ada*, Ahcy, *Alpk1*, *Armc6*, *Batf*, *Bcl2a1a*, *Bcl2a1d*, *Bst1*, *Ccl2*, *Ccl24*, *Ccl3*, *Ccl4*, *Ccl7*, *Ccnb2*, *Ccr1*, *Cd109*, *Cd14*, *Cd24a*, Cd300lf, *Cd86*, *Cdk6*, *Clec4n*, *Clec5a*, *Col3a1*, *Colec12*, *Csf1*, *Ctse*, *Cx3cr1*, *Cxcl14*, *Fam20c*, *Glo1*, *Gm8909*, *Gpr183*, *H2-Ab1*, *H2-DMb1*, *H2-K1*, *H2-L*, *H2-Q1*, *H2-Q2*, *H2-Q4*, *H2-Q6*, *H2-Q8*, *H2-Q9*, *H2-T22*, *H2-T24*, *Hist1h2bf*, *Hist1h2bk*, *Hist1h2bl*, *Hist2h3c2*, *Hist1h3a*, *Hist1h3b*, *Hist1h3d*, *Hist1h3g, Hist1h3h*, *Hist1h3i*, *Hist1h4a*, *Hist1h4f*, *Hist1h4i*, *Hist4h4*, *Ifitm1*, *Ifitm3*, *Il1rn*, *Irf7*, *Kdr*, *Lat2*, *Malt1*, *Mmp14*, *Myc*, *Ndrg1*, *Npy*, *Oasl1*, Pla2g7, *Ripk3*, *Serpine1*, *Slpi*, *Spn*, *Spp1*, *Stap1*, *Tnfsf13*, *Tnfsf8*, *Top2a*	2.88e^−79^	1.00e^−76^
BALB/c_*La vs*. BALB/c	*Il1b*, *Mef2c*	*Cxcl1*, *Cxcl2*, *Cxcl3*, *Hilpda, Id2*, *Irg1*, *Smad6*, *Tnfrsf26*	2.43e^−8^	2.24e^−6^
C57BL/6_*La vs*. C57BL/6	*Ccr2*, *Ccr5*, *Fcgr1*, *Foxo3*, *Gcnt1*, *Gpr183*, *Hhex*, *Hist1h2bc*, *Hist1h2be*, *Hist1h2bg*, *Hist1h3e*, *Hist1h4c*, *Hist1h4d*, *Hist1h4h*, *Hist1h4m*, *Hist2h3b*, *Hist2h4*, *Il16*, *Lyl1*, *Mafb*, *Mapk14*, *Mef2c*, *Mertk*, *Mtus1*, *Pik3cd*, *Rassf2*, *Themis2*, *Tlr8*, *Tnfaip8l2*, *Trim14*, *Tsc22d3*, *Zfp36l1*, *Zfp36l2*	*Adora2b*, *Ampd3*, *Batf*, *Bcl2a1a*, *Bcl2a1d*, *Bcl3*, *Birc3*, *Ccl3*, *Ccl4*, *Cd24a*, *Cd274*, *Cd40*, *Cd83*, *Cd86*, *Cdkn1a*, *Cdkn2b*, *Cebpb*, *Clec4d*, *Clec4e*, *Clec5a*, *Cxcl1*, *Cxcl2*, *Cxcl3*, *Ednrb*, *Ezr*, *Fam20c*, *Fas*, *Gadd45g*, *Gbp5*, *Gch1*, *Gpr68*, *H2-M2*, *Hcar2*, *Hilpda*, *Hmox1*, *Hsp90aa1*, *Hyal2*, *Icam1*, *Icosl*, *Id2*, *Il17ra*, *Il1rn*, *Il27*, *Irak2*, *Irf1*, *Irg1*, *Jag1*, *Jun*, *Lcp2*, *Lilrb4a*, *Malt1*, *Mb21d1*, *Mefv*, *Mmp14*, *Nck1*, *Nfe2l2*, *Nfkb1*, *Nfkb2*, *Nfkbia*, *Nlrp3*, *Nod2*, *Nr1h3*, *Olr1*, *Osm*, *Pde4b*, *Pmaip1*, *Ppp4r2*, *Prdx1*, *Procr*, *Ptafr*, *Rbpj*, *Rgcc*, *Ripk2*, *Rnf19b*, *Samsn1*, *Serpine1*, *Sh2b2*, *Slamf7*, *Slc11a2*, *Smad6*, *Sod2*, *Sqstm1*,*Src*, *Stx11*, *Tiparp*, *Tlr2*, *Tnf*, *Tnfaip3*, *Tnfrsf26*, *Tnfsf9*, *Tnip1*, *Tnip3*, *Traf3*, *Trim13*	1.40e^−65^	2.37e^−63^
C57BL/6_*La vs*. BALB/c_*La*	*Adgre1*, *Ampd3*, *Ang*, *Apobec3*, *Axl*, *Bcl3*, *Bcl6*, *Birc3*, *Blnk*, *Bloc1s6*, *C1qa*, *C1qb*, *C1qc*, *C5ar1*, *Camp*, *Casp1*, *Ccl3*, *Ccl4*, *Ccl5*, *Ccl9*, *Ccr2*, *Ccr3*, *Cd274*, *Cd38*, *Cd4*, *Cd40*, *Cd79b*, *Cd83*, *Cdkn1a*, *Cebpb*, *Clec1b*, *Clec2d*, *Clec2i*, *Clec4e*, *Cnr2*, *Ctsh*, *Cxcl1*, *Cxcl10*, *Cxcl2*, *Cxcl3*, *Cxcl9*, *Ednrb*, *Fas*, *Fcgr4*, *Fcna*, *Fyb*, *Fzd7*, *Gbp2*, *Gbp3*, *Gbp5*, *Gbp6*, *Gbp7*, *Gpr68*, *H2-Ab1*, *H2-DMb1*, *H2-K1*, *H2-L*, *H2-M2*, *Hmox1*, *Icam1*, *Icosl*, *Iigp1*, *Il10*, *Il17ra*, *Il18bp*, *Il1a*, *Il1f9*, *Il27*, *Irak2*, *Irak3*, *Irf1*, *Irg1*, *Itgad*, *Itgal*, *Jag1*, *Kdr*, *Lcn2*, *Lcp2*, *Malt1*, *Mapkapk2*, *Marco*, *Mefv*, *Mertk*, *Mill2*, *Nfkb1*, *Nfkb2*, *Nfkbia*, *Nfkbid*, *Nlrc4*, *Nlrp3*, *Nod1*, *Nr1h3*, *Olr1*, *Pde4b*, *Pmaip1*, *Pnp*, *Ppp4r2*, *Prdm1*, *Procr*, *Ptafr*, *Ptprj*, *Rab32*, *Rela*, *Relb*, *Rgcc*, *Ripk2*, *Rnf19b*, *S100a8*, *Sh2b2*, *Skil*, *Slamf7*, *Slc40a1*, *Smad6*, *Snx10*, *Sod2*, *Sqstm1*, *Stx11*, *Tapbpl*, *Tbk1*, *Tgtp1*, *Thbs1*, *Tlr1*, *Tlr2*, *Tmem176a*, *Tmem176b*, *Tnf*, *Tnfaip3*, *Tnfrsf1b*, *Tnfsf9*, *Tnip3*, *Traf3*, *Treml4*, *Trib1*, Trim13, *Vcam1*, *Vegfa*, *Vsig4*	*Ada*, *Ahcy*,*Aim2*, *Alpk1*, *Armc6*, *Bst1*, *Ccl2*, *Ccl24*, *Ccl7*, *Ccnb2*, *Cd109*, *Cd300lf*, *Cfb*, *Clec4n*, *Csf1*, *Colec12*, *Col3a1*, *Ctse*, *Cxcl14*, *Gcnt1*, *Glo1*, *Gm8909*, *Gpr183*, *H2-Q1*, *H2-Q2*, *H2-Q6*, *H2-Q8*, *H2-Q9*, *H2-T22*, *H2-T24*, *Hhex*, *Hist1h2ba*, *Hist1h2be*, *Hist1h2bf*, *Hist1h2bg*, *Hist1h2bk*, *Hist1h2bl*, *Hist1h3a*, *Hist1h3b*, *Hist1h3d*, *Hist1h3e*, *Hist1h3h*, *Hist1h3i*, *Hist1h3g*, *Hist1h4a*, *Hist1h4b*, *Hist1h4d*, *Hist1h4f*, *Hist1h4h*, *Hist1h4i*, *Hist1h4j*, *Hist1h4k*, *Hist1h4m*, *Hist1h4n*, *Hist2h3b*, *Hist2h3c2*, *Hist2h4*, *Hist4h4*, *Ifitm1*, *Ifitm3*, *Il16*, *Il1rn*, *Irf4*, *Irf7*, *Junb*, *Lgals1*, *Mmp9*, *Ndrg1*, *Npy*, *Pdgfrb*, *Pla2g7*, *Ripk3*, *Serpine1*, *Slfn1*, *Slpi*, *Spn*, *Spp1*, *Tacc3*, *Tnfsf13*, *Tnfsf8*, *Top2a*,	2.02e^−125^	9.61e^−123^

Gene Ontology (GO) enrichment analysis and the profiling of differentially expressed genes (DEGs) involved in the immune system processes in bone marrow-derived macrophages (BMDMs) from BALB/c and C57BL/6 in response to *L. amazonensis* (*La*) infection. The analysis was based on *p*-values and false discovery rates (FDRs).

**Figure 3:**
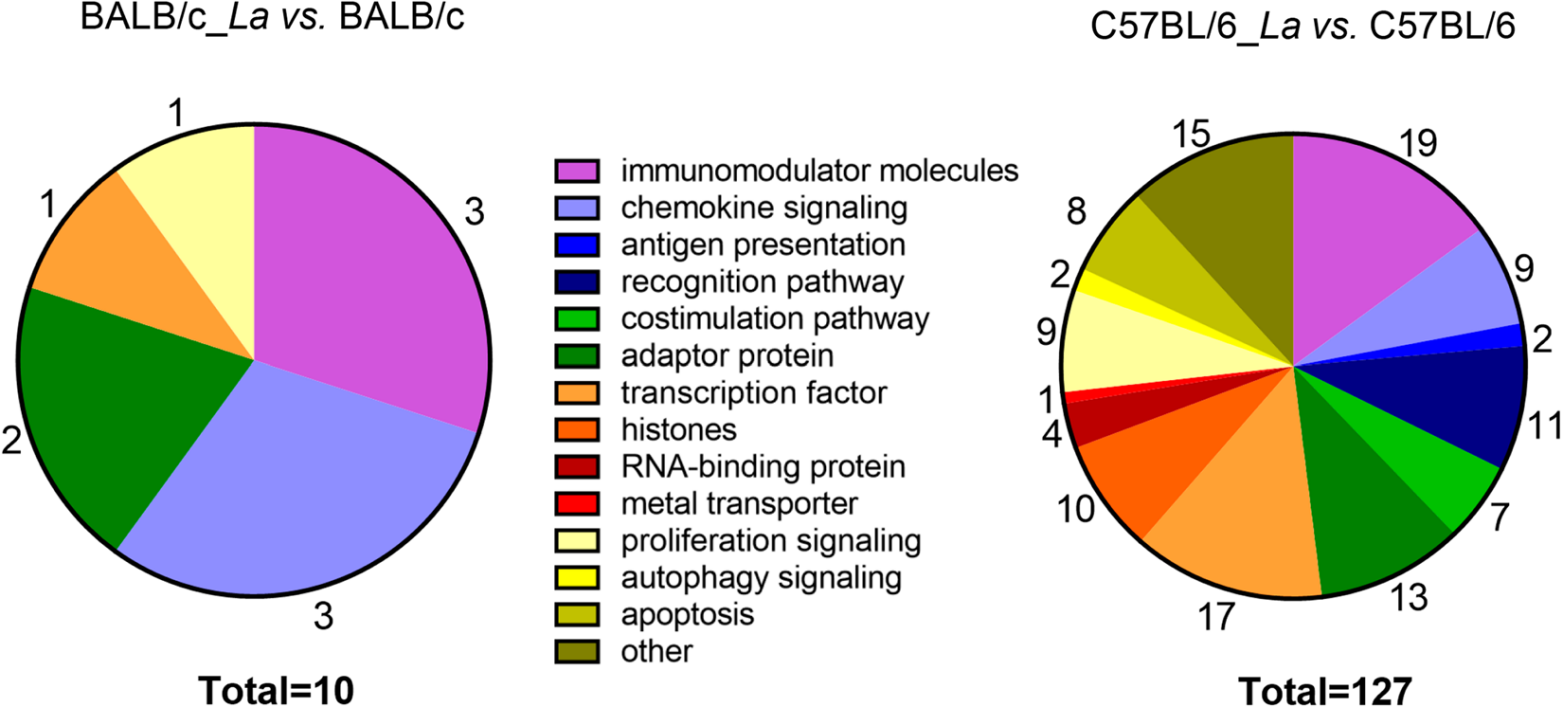
Immune response analysis of DEGs in BMDMs from BALB/c and C57BL/6 mice in response to *L. amazonensis* infection. Pie chart of the modulated molecules involved in the immune response processes grouped into main immune signaling pathways.

Furthermore, we categorized the identified molecules according to the main types of immune system processes in response to *L. amazonensis* infection (Fig. 3). In the BALB/c_*La vs*. BALB/c comparison, we found that most of the modulated transcripts were immunomodulatory (*Il1b*, *Irg1* and *Tnfrsf26*) and chemokine signaling molecules (*Cxcl1*, *Cxcl2* and *Cxcl3*) (Fig. 3 and Table 1). In the C57BL/6_*La vs*. C57BL/6 comparison, most of the modulated transcripts were immunomodulatory molecules (*Clec4d*, *Clec4e*, *Clec5a*, *Il16*, *Il17ra*, *Il1rn*, *Il27*, *Irak2*, *Irg1*, *Lcp2 Mefv*, *Themis2*, *Tnf*, *Tnfaip3*, *Tnfaip8l2*, *Tnfrsf26*, *Tnfsf9*, *Tnip1* and *Tnip3*), transcription factors (*Batf*, *Bcl3*, *Cebpb*, *Foxo3*, *Hhex*, *Id2*, *Irf1*, *Jun*, *Lyl1*, *Mafb*, *Nfkb1*, *Nfkb2*, *Nfkbia*, *Tiparp*, *Trim13*, *Trim14* and *Tsc22d3*), adaptor proteins (*Malt1*, *Mef2c*, *Nck1*, *Nfe2l2*, *Nr1h3*, *Pik3cd*, *Procr*, *Ptafr*, *Rbpj*, *Rgcc*, *Sh2b2*, *Smad6* and *Src*) and members of recognition pathways (*Birc3*, *Jag1*, *Lilrb4a*, *Mapk14*, *Mb21d1*, *Nlrp3*, *Nod2*, *Ripk2*, *Tlr2*, *Tlr8* and *Traf3*) (Fig. 3 and Table 1).

### The exclusive differential gene expression patterns in BMDMs from C57BL/6 mice appeared to be mostly related to proliferation signaling and transcription factor molecules

**Figure 4:**
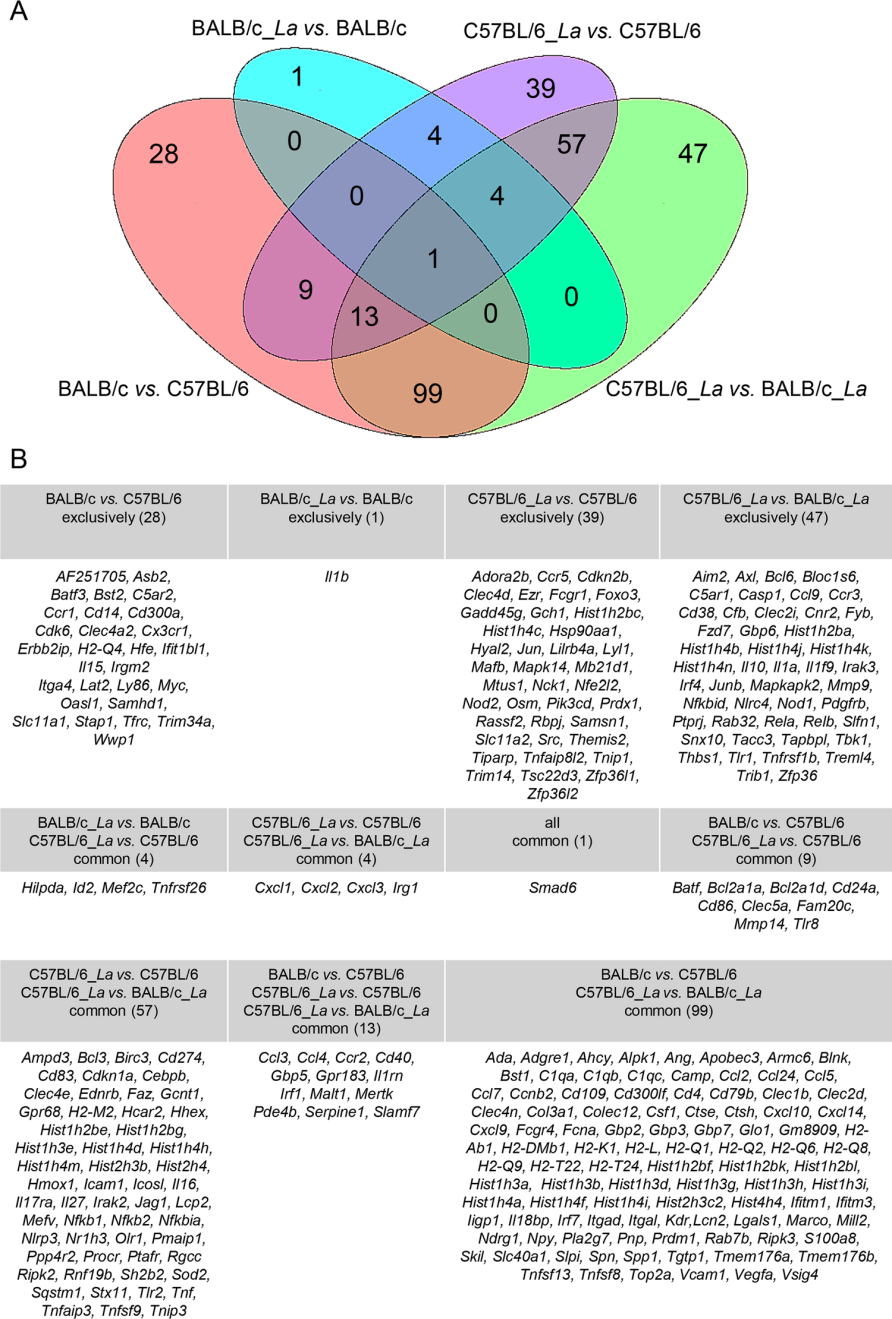
Venn diagram analysis of DEGs in BMDMs from BALB/c and C57BL/6 mice in response to *L. amazonensis* infection. (**A**) Venn diagram of the 361 DEGs involved in the immune response processes, showing the numbers of exclusively and common genes for each comparison. (**B**) List of exclusively and common genes according for each comparison in the Venn diagram.

Venn diagram analysis was performed, and based on the results, we grouped the exclusively and commonly modulated genes involved in the immune response. We identified 28 exclusively modulated genes in the comparison of the two host backgrounds (in non-infected macrophages). Additionally, we identified only one exclusively modulated gene in BALB/c_*La vs*. BALB/c macrophages, 39 exclusively modulated genes in C57BL/6_*La vs*. C57BL/6 macrophages, and 47 exclusively modulated genes in C57BL/6_*La vs*. BALB/c_*La* macrophages. Interestingly, only one gene, *Smad6*, was common among all comparisons (Fig. 4A).

**Figure 5:**
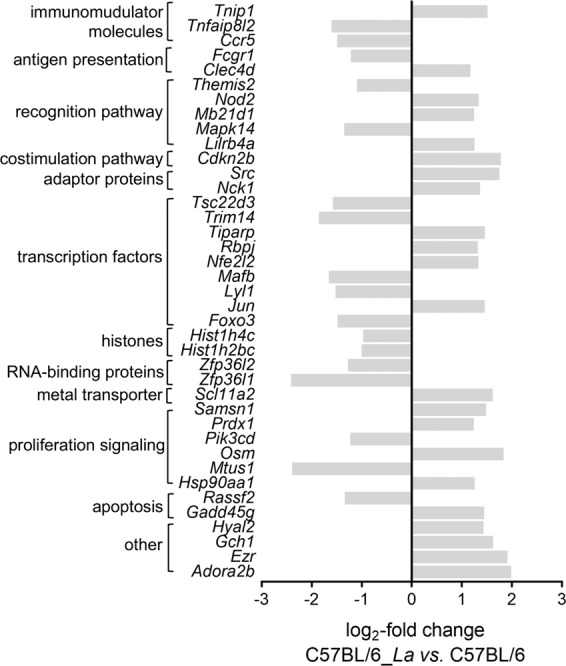
DEGs profile of the exclusively modulated genes involved in the immune response processes in infected C57BL/6 *vs*. non-infected C57BL/6 BMDMs. The profiles of DEGs are presented as the log_2_-fold changes in the expression of the 39 exclusively modulated genes involved in the immune response processes in BMDMs from C57BL/6 infected with *L. amazone*nsis *vs*. non-infected C57BL/6 BMDMs. The genes were classified by their involvement in main immune response signaling pathways or by their identities as regulatory molecules of the immune response pathways. La, *L. amazonensis*.

Examination of the immune response modulation associated with *L. amazonensis* infection and the pattern of exclusively expressed genes in the comparison of BALB/c_*La vs*. BALB/c macrophages revealed the downregulation of *Il1b* as unique (Fig. 4B). In contrast, comparison of C57BL/6_*La vs*. C57BL/6 macrophages revealed a set of 39 modulated genes, among which 22 were upregulated genes and 17 were downregulated. Most of these genes appeared to be involved in the proliferation pathway, such as the downregulated *Pik3cd* and *Mtus1* genes and the upregulated *Samsn1*, *Prdx1*, *Osm* and *Hsp90aa1* genes. Another group of genes contained transcription factors, including the downregulated *Tsc22d3*, *Trim14*, *Mafb* and *Lyl1* genes and the upregulated *Tiparp*, *Rbpj*, *Nfe2l2*, *Jun* and *Foxo3* genes. We also identified genes involved in recognition and costimulation pathways, as well as genes encoding adaptor molecules: *Fcgr1*, *Mapk14* and *Themis2* were downregulated, while *Mb21d1/cGas*, *Nod2, Clec4d*, *Lilrb4a*, *Cdkn2b, Nck1, Src* and *Tnip1* were upregulated. Among apoptosis-related molecules, *Rassf2* and *Gadd45g* appeared downregulated. The immunomodulatory molecules *Tnfaip8l2* and *Ccr5* were downregulated. The metal transporter *Slc11a2* (formerly *Nramp2*) was upregulated. The histones *Hist1h2bc* and *Hist1h4c*, as well as the RNA-binding proteins *Zfp36l1* and *Zfp36l2*, were downregulated. Poorly studied molecules, such as *Adora2b*, *Ezr*, *Gch1* and *Hyal2* were upregulated and were classified as belonging to other pathways (Fig. 5).

**Figure 6:**
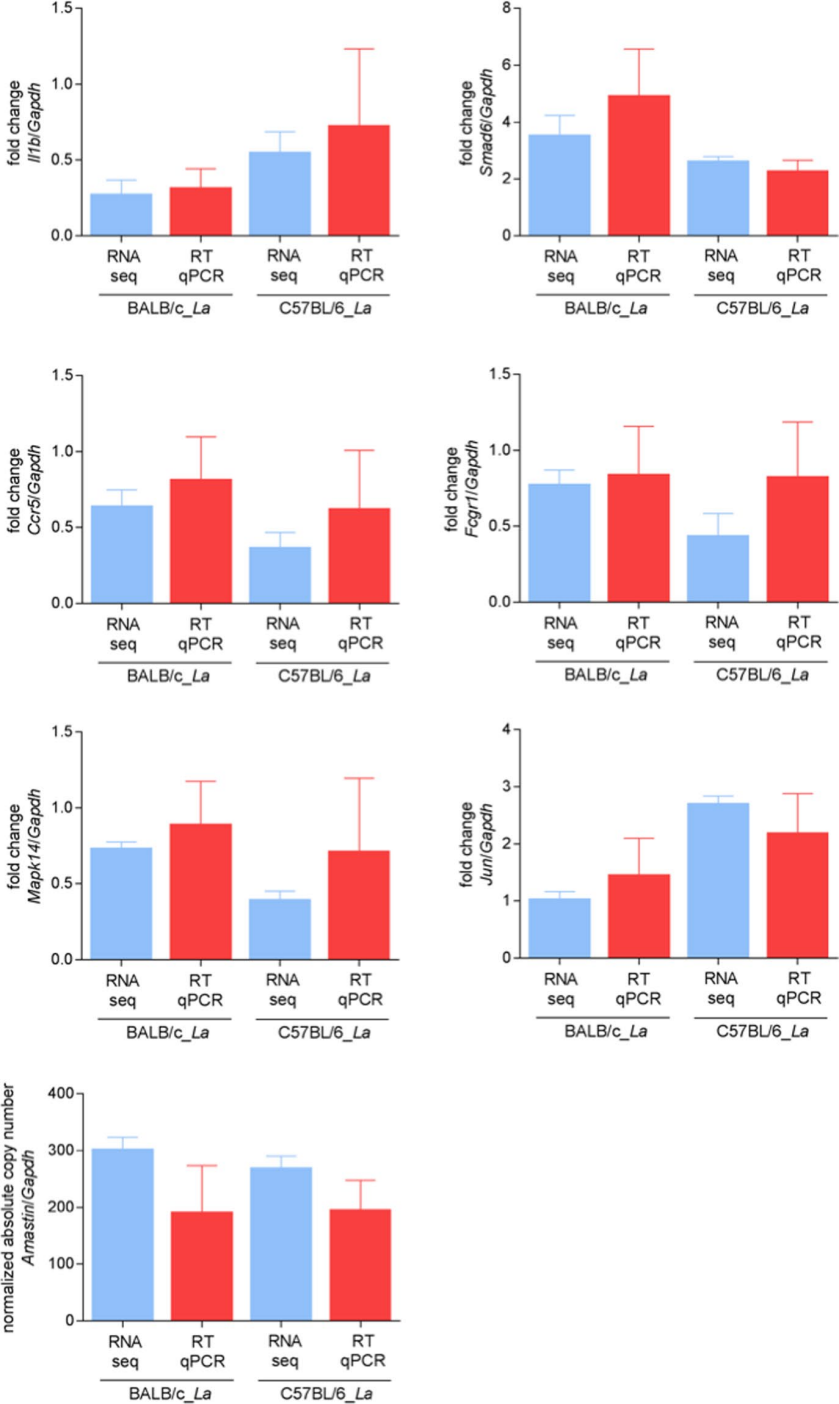
RT-qPCR validation of some modulated genes in BALB/c and C57BL/6 BMDMs in response to *L. amazonensis* infection. Comparative analysis of the relative expression levels of selected genes determined by RNA-seq and validated by RT-qPCR. The bars represent the mean ± SD values of the fold changes in *Il1b*, *Fcgr1*, *Ccr5*, *Smad6*, *Jun* and *Mapk14* expression determined with five independent biological replicates analyzed in duplicate. The fold changes were calculated through relative quantification using the ΔΔCt method. The data were normalized to *Gapdh* expression and the relative gene expression was set to 1 for the control (non-infected) samples. Statistical analysis was performed using the *t*-tests, and no significant differences were observed (*p*-value < 0.05) between the RT-qPCR and RNA-seq results for the BALB/c_*La* and C57BL/6_*La* groups. The bars for *Amastin*-like (LmxM.33.0960) show the mean after normalization to *Gapdh* in *L. amazonensis* infecting BALB/c and *L. amazonensis* infecting C57BL/6 macrophages. La, *L. amazonensis*.

RT-qPCR validation assays were performed on some of the most modulated molecules from the RNA-seq data: *Il1b*, *Fcgr1*, *Ccr5*, *Smad6*, *Jun* and *Mapk14*. Comparative analyses showed concordance between the RNA-seq and RT-qPCR data with no statistically significant differences, thus validating the RNA-seq results (Fig. 6).

Similar to BMDMs, peritoneal macrophages were collected and infected with *L. amazonensis*, and the gene expression modulation of selected genes was analyzed by RT-qPCR to evaluate whether a similar trend occurred in another macrophage subtype. The infection index appeared significantly lower in C57BL/6_*La* macrophages than in BALB/c_*La* macrophages (Fig. S3A), indicating a distinct phenotypic difference between BMDMs and peritoneal macrophages in response to *L. amazonensis* infection. Comparison of the gene expression in BMDMs and peritoneal macrophage subtypes from BALB/c_*La* mice revealed lower expression of *Smad6* and *Mapk14*. No modulation of *Il1b*, *Ccr5*, *Fcgr1* or *Jun* was observed. On the other hand, we observed lower expression of *Smad6*, higher expression of *Ccr5* and no modulation of *Il1b*, *Mapk14*, *Fcgr1* and *Jun* expression in BMDMs compared with peritoneal macrophage subtype from C57BL/6_*La* (Fig. S3B).

**Figure 7:**
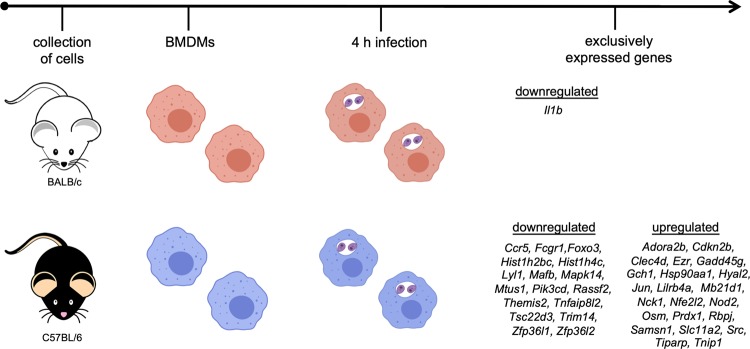
Schematic representation of the exclusive genes and DEGs in BALB/c and C57BL/6 BMDMs in response to *L. amazonensis* infection. Summary of the data of the exclusive genes and DEGs in BMDMs derived from BALB/c and C657BL/6 mice in response to early *L. amazonensis* infection.

The transcriptomic data presented here corroborate the findings of previous studies on how differential genetic backgrounds from different hosts define susceptibility or resistance to *Leishmania* infection. The DEGs profiles described in this work represents new knowledge obtained from transcriptome analyses of immune responses between two different host genetic backgrounds. The analyses identified molecular markers that could be linked to susceptibility and resistance to *L. amazonensis* infection, as illustrated by the schematic representation of the exclusively and DEGs in BMDMs from BALB/c and C57BL/6 mice in response to early *L. amazonensis* infection (Fig. 7).

### Parasite transcriptome profiling revealed only one DEG between L. amazonensis infecting BALB/c and L. amazonensis infecting C57BL/6 macrophages

We also analyzed the gene expression of *L. amazonensis* via alignment to the *L. mexicana* genome database (Table S1). The sequencing data are available in the NCBI BioProject and SRA databases, as previously described.

After initial assembly, 8,282 parasite transcripts were identified. Analysis of DEGs with significant threshold of a fold change ≥ 2 and a *p*-value < 0.05, as statistically significant, revealed only one DEG, a noncoding RNA (ncRNA) (LmxM.32.ncRNA:rfamscan:912871–912976), which showed higher expression in infected BALB/c than in infected C57BL/6 macrophages.

Additionally, we performed RT-qPCR validation assays of our RNA-seq data for *Amastin*-like gene (LmxM.33.0960). Similar to the case for the host comparative analyses, we observed concordance between the RNA-seq and RT-qPCR data (Fig. 6), thus validating the RNA-seq results.

Finally, we observed lower expression of the *Amastin*-like gene (LmxM.33.0960) in peritoneal macrophages than in BMDMs from BALB/c and C57BL/6 mice (Fig. S3B).

## Discussion

The Th1/Th2 paradigm correlating resistance/susceptibility to *Leishmania* infection has been extensively studied^3,14,18–21,23,35,41^. Identification of potential biomarkers for leishmaniases can be useful for different approaches, such as diagnosis, prognosis, disease progression monitoring, clinical intervention and host immune response characterization^33,34,41,43–45^. The host-parasite interaction depends on both host genetic backgrounds^33,35,36,41^ and the genetic complexity of *Leishmania* species^39,40,46^.

*L. amazonensis* infection elicits different immune responses than those previously described for *L. major* infection^25,26,28,29,31,35^. In this work, we present the global transcriptome profiles of BMDMs from BALB/c and C57BL/6 mice non-infected and infected with *L. amazonensis*, focusing on the modulation of the immune response. In the absence of *L. amazonensis* infection, we identified significantly different basal gene expression patterns between the two hosts, corroborating with previous findings^47^. Analysis of the immune response in early *L. amazonensis* infection revealed 361 modulated genes among the comparisons. Comparison of infected BALB/c to non-infected BALB/c BMDMs revealed low levels of gene expression modulation; this pattern could be related to limited immune response activation, leading to susceptibility of this host to *L. amazonensis* infection, as previously described^21,48^. The DEGs involved in immune response modulation comprised mostly immunomodulatory and chemokine signaling molecules, suggesting a link to the inflammation process. In contrast, we observed high levels of gene expression modulation in infected C57BL/6 compared to non-infected C57BL/6 BMDMs. This pattern could be related to increased immune response activation via augmentation of recognition processes and, consequently, activation of signaling cascades, leading to moderate resistance of this host to *L. amazonensis* infection. Different profiles associated with different host genetic backgrounds have previously been described as being due to different parasite burdens, inflammatory cell populations and cytokine production^21,48^.

The infection index of BMDMs from BALB/c mice appeared smaller than that of BMDMs from C57BL/6 mice after 4 h of infection. As the infection index represents the number of intracellular parasites multiplied by the percentage of infected macrophages, the biological impact of this difference indicates that at an early stage of infection, C57BL/6 macrophages exhibit greater phagocytosis, which in subsequent times of infections may enable control of parasite replication. Previous studies by our group have demonstrated increased infection index values in BALB/c macrophages after 24 and 48 h of infection; in contrast, the index values of C57BL/6 macrophages appeared to remain stable^49,50^. However, most gene expression modulation has been described to occur during early *Leishmania* infection^33,38,41,50,51^.

The fact that *Il1b* appeared to be downregulated and was an exclusively modulated gene involved in the immune response in infected BALB/c compared to non-infected BALB/c BMDMs corroborates the important role of this molecule in *Leishmania* infection. IL1β has previously been identified as an important signaling factor for host resistance to C57BL/6 infection, since this cytokine signals through IL1R and MyD88 to induce NOS2-mediated NO production, which is a major host defense mechanism against *Leishmania*^52^. Furthermore, polymorphisms in the *Il1b* gene are associated with the severity of the disease in patients infected with *L. mexicana*^53^. Given these findings, we reinforce the importance of this molecule in *Leishmania* infection in both hosts^52–54^.

The 39 exclusively modulated immune response-related genes in infected C57BL/6 compared to non-infected C57BL/6 BMDMs were associated with important signaling pathways, suggesting enhancement of immune response activation resulting in moderated resistance against *L. amazonensis* infection. The recognition signaling cascade included a large number of modulated molecules, highlighting the importance of the host genetic background in the initial steps of macrophage activation^55^. Among these molecules, NOD-like receptors play protective roles during *Leishmania* infection^52,56,57^. The upregulation of *Nod2* in infected C57BL/6 compared to non-infected BMDMs indicates greater macrophage activation in C57BL/6 mice. NOD2 mediates the parasite-induced production of cytokines, such as IL-17 and IFN-γ production, in *L. infantum* and *L. amazonensis* infections, whereas NOD1 is not relevant to these infections^56,57^. Recognition signaling also involves MAPKs, which play important roles against parasitic infections^58^, driving the switch in macrophage activation from proinflammatory IL12 to anti-inflammatory IL10 cytokines^59^. Previous studies have demonstrated that signaling during *L. amazonensis* infection leads to the activation of MAPK1 and MAPK3^58^. MAPK14 has been poorly studied in the context of *Leishmania* infection, although downregulation of *Mapk14* has previously been described to occur in *L. braziliensis* and *L. major* infections^41,60^. Most molecules from the recognition pathway were upregulated, indicating the activation of the downstream steps in recognition signaling cascades.

Immunomodulatory molecules play important roles in macrophage activation and the induction of adaptive immune responses via cytokine production in response to *Leishmania* infection^13,20,61^. Among the main cytokines studied, TNF is a multipotent cytokine implicated in a wide range of immune responses occuring in response to many infections^62,63^. In particular, the TNF-related molecules *Tnip1* and *Tnfaip8l2* appeared upregulated and downregulated, respectively, indicating signaling cascade activation and repression to maintain immune homeostasis. *Leishmania* infection can also induce the expression of numerous chemokines^26,51,64,65^. This event could potentially benefit the parasite due the ability to repress the induction of proinflammatory cytokine expression^66^. The downregulation of *Ccr5* in infected C57BL/6 *vs*. non-infected C57BL/6 BMDMs could be correlated with the fact that this receptor directs the Th1 immune response and is thus associated with inflammation, cell infiltration and the development of infectious disease^67^. Previous studies have demonstrated that CCR5 knockout mice exhibit increased resistance to *L. major* infection^68^.

Similarly, human macrophage infections with *L. amazonensis*, *L. major* and *L. panamensis* have been shown to elicit immune response modulation of TNF, NF-kB and NOD-like receptor signaling pathways, oxidative stress pathways and proliferation signaling pathways^41,69^.

The expression of proliferation signals and transcription factor-related molecules was highly modulated according to our data. There are limited descriptions of these molecules; however, they are known to control the expression of many genes required for the effective activation of the immune responses, such as transcriptional activators or repressors, as well as for FOXO transcriptional activity, NF-kB recruitment and Notch signaling^70–73^.

The release and activation of histones occur in response to stress, leading to Toll-like receptor binding and triggering the activation of multiple signaling pathways^74^. The downregulation of *Hist1h4c* and *Hist1h2bc* could be related to the negative modulation of transcription factors listed above that are involved in macrophage activation.

The metal transporter natural resistance-associated macrophage protein (Nramp) has been associated with resistance to intracellular pathogens due to enhanced NOS2 expression and NO production^75,76^. Point mutations in *Nramp1* promote susceptibility to *Leishmania* infection by modulating iron acquisition from intracellular compartments^76,77^, starving pathogens of this essential nutrient and impacting parasite survival and replication^78^. Although *Nramp2* shares a conserved structure and iron transport functions with *Nramp1*, its role in *Leishmania* infection has been poorly studied. The upregulation of *Slc11a2* (formerly *Nramp2)* was upregulated in infected C57BL/6 macrophages could be correlated with increased NO production and resistance to *L. amazonensis* infection.

Apoptosis induced by *Leishmania* may permit successful infection through modulation of host immunity^79^. RASSF2 and GADD45G are involved in the regulation of growth and apoptotic processes. Consistent with these findings, we identified downregulation of *Rassf2* and the upregulation of *Gadd45g* as important factors in the modulation of the host immunity in response to *L. amazonensis* infection.

Molecules not acting in any of the described pathways were classified as “other” due to their limited descriptions in *Leishmania* infection. Further studies and functional validation could implicate the role of these molecules in host immune modulation in response to *L. amazonensis* infection, but this study provides only a global transcriptomic view based on the profile of the DEGs involved in immune response modulation in the two different host genetic backgrounds.

Macrophages form a vast and diverse population with considerable plasticity to adapt to different tissues and change in response to environmental variations^80–83^. The differences between peritoneal macrophages and BMDMs are believed to arise from differential physiological conditions and organ specificity along with the heterogeneity of macrophages^83,84^. Thus, we compared BMDMs and peritoneal macrophages with regard to some of the modulated genes to reinforce our findings and provide a representation of the *in vivo* scenario. According to our results, the infection index appeared lower in peritoneal macrophages from C57BL/6 mice than in those from BALB/c mice, indicating a distinct phenotypic difference between the macrophage subtypes in response to early *L. amazonensis* infection and suggesting that BALB/c mice are more susceptible models than C57BL/6 mice, as previously described^21,48^. Analyses of gene expression have shown a similar gene expression profiles in the comparison of BMDMs and pre-existing populations, although some differences have also been reported, suggesting that tissue environments dictate the macrophage phenotype required to trigger an effective immune response^80,81^. In our comparisons we observed nondifferential and differential modulation patterns, indicating that some of the analyzed genes were involved in distinct signaling cascades that lead to a distinct network activity. *Smad6* showed a lower gene expression pattern in peritoneal macrophages than in BMDMs in both BALB/c and C57BL/6 mice. Since *Smad6* is a regulator of myeloid differentiation^85^, this expression pattern confirms the differences between the macrophage subtypes. There were no differences in the gene expression patterns of *Jun*, *Fcgr1* and *Il1b* between macrophage subtypes, suggesting similar trends of activation of transcription factor binding, recognition, phagocytosis and proinflammatory cytokines production. *Ccr5* showed high modulation only in peritoneal macrophages from infected C57BL/6 mice, indicating upregulation of this chemokine receptor in this macrophage subtype. *Mapk14* showed low modulation only in peritoneal macrophages from infected BALB/c mice, indicating low activation of the cellular response cascade in this macrophage subtype.

Altogether, our findings indicate the need to be cautious in extrapolating findings to *in vivo* scenarios that may or may not differ from those observed in the present study, especially considering that other immune cells, such as monocytes, neutrophils and lymphocytes, migrates to local cutaneous lesions with *Leishmania*^86–88^. Both host and parasite genetic backgrounds also need to be considered in translational approaches to identify biomarkers for the prognosis determination and treatment of the leishmaniases.

Finally, the transcriptome profiling of the parasite revealed only one DEG between *L. amazonensis* infecting BALB/c macrophages and *L. amazonensis* infecting C57BL/6 macrophages, a noncoding RNA (LmxM.32.ncRNA:rfamscan:912871–912976). ncRNAs have several functions; for example, they mediate transcription by RNA polymerase II, polyadenylate 3´-ends, regulate transcript expression and are potentially associated with small ribonucleoprotein complexes^89^. Our observations indicate that during early infection, the parasite exhibits the same gene expression pattern regardless of the host genetic background.

## Methods

### Animals

Female BALB/c and C57BL/6 mice (6–8 weeks old) were obtained from the Animal Center of the Medical School of the University of São Paulo and were maintained at the Animal Center of the Department of Physiology of the Institute of Bioscience of the University of São Paulo with access to food and water *ad libitum*.

### Leishmania culture

*L. amazonensis* (MHOM/BR/1973/M2269) was grown at 25 °C in M199 medium (Gibco, Grand Island NY, USA), pH 7.0, supplemented with L-glutamine, 10% heat-inactivated fetal bovine serum, 0.25% hemin, 40 mM NaHCO_3_, 100 μM adenine, 40 mM HEPES, 100 U/mL penicillin and 100 μg/mL streptomycin, as previously described^37–39^. The parasites were counted in a Neubauer chamber.

### *In vitro* macrophage infections

BMDMs were obtained from the femurs of BALB/c and C57BL/6 mice through PBS washing, and the cells were collected by centrifugation at 500 x g for 10 min at 4 °C. Lysis of erythrocytes was performed with NH_4_Cl (145 mM) and Tris-base (200 mM), pH 7.0, followed by incubation on ice for 20 min. After lysis, the cells were washed with cold PBS, centrifuged at 500 x g for 10 min at 4 °C and incubated in RPMI 1640 medium supplemented with penicillin (100 U/mL), streptomycin (100 µg/mL), 2-mercaptoethanol (50 µM), L-glutamine (2 mM), sodium pyruvate (1 mM), 10% fetal bovine serum and 10% L929 conditioned medium as a macrophage stimulating factor source. The cells were differentiated for 7 days at 34 °C in 5% CO_2_. The BMDMs were used after phenotypic analysis by flow cytometry showed at least 95% F4/80 and CD11b-positive cells, as previously described^50^. After macrophage differentiation, cellular viability was evaluated with Trypan blue staining (1:1 (v:v)), and the cells were counted in a Neubauer chamber. Approximately 5 × 10^6^ BMDMs from BALB/c and C57BL/6 mice were incubated in sterile 6-well plates (SPL Life Sciences, Korea) overnight at 34 °C in 5% CO_2_. Non-adherent cells were removed by washing with PBS, and infection was performed with *L. amazonensis* promastigotes in the stationary growth phase (MOI 5:1). After 4 h of infection, the cultures were washed with PBS; then, RNA was extracted, or the infection index was determinated. Non-infected macrophages maintained in culture under the same conditions were used as the controls. The infections were evaluated by determining the percentage of infected cells after counting 400 panoptic-stained (Laborclin, Parana, Brazil) macrophages. The infection index was determined by multiplying the percentage of infected macrophages by the mean number of intracellular parasites per infected cell^90,91^. Statistical analyses were performed using Student´s *t*-test and *p*-value < 0.05 was considered to indicate a significant difference between infected C57BL/6 macrophages or infected BALB/c macrophages and the corresponding non-infected macrophages.

Peritoneal macrophages were collected from BALB/c and C57BL/6 mice by injection and recovery of 5 mL of RPMI 1640 medium supplemented, as previously described. The cells were recovered by centrifugation at 500 × g for 10 min at 4 °C. Cellular viability was evaluated with Trypan blue staining (1:1 (v:v)), and the cells were counted in a Neubauer chamber. Approximately, 1 × 10^6^ peritoneal macrophages were incubated in sterile 6-well plates (SPL Life Sciences, Korea) overnight at 34 °C in 5% CO_2_. Non-adherent cells were removed by washing with PBS, and infection was performed with *L. amazonensis* promastigotes in the stationary growth phase (MOI 5:1). After 4 h of infection, cultures were washed with PBS; then, RNA was extracted or the infection index was determined. Non-infected macrophages maintained in culture under the same conditions were used as the controls. The infections were evaluated as previously described for BMDMs.

### Total RNA isolation and library construction

Total RNA was isolated from five independent biological replicates of each infected and non-infected group using TRIzol reagent (Life Technologies, Carlsbad, CA, USA) according to the manufacturer’s instructions and as previously described^39^. The RNA samples were treated with DNase I (1 U per µg of RNA) (Thermo Scientific, Lithuania, EU) at 37 °C for 1 h, and the RNA concentration was determined from the A260/A280 ratio using a NanoDrop ND1000 (Thermo Scientific, USA). In addition, RNA integrity was evaluated using an Agilent 2100 Bioanalyzer and a Pico Agilent RNA 6000 kit (Agilent Technologies, Santa Clara, CA, USA) according to the manufacturer’s instructions. rRNA depletion was performed using a poly(A) magnetic bead capture protocol and a TrueSeq Stranded Total RNA Sample Prep kit (Illumina) according to the manufacturer´s instructions. Libraries were prepared using a TrueSeq Stranded RNA-seq Library Prep Kit (Illumina), according to the manufacturer’s instructions.

### RNA-seq and data analysis

Paired end reads (100 bp) were obtained using an Illumina NovaSeq. 6000 platform at Macrogen Inc. (Seoul, South Korea). Quality control was performed on the sequenced raw reads based on the read quality, total bases, total reads, GC content (%) and basic statistics. The quality of the reads was analyzed using FastQC according to the Phred quality score^92^. Reads with Phred quality scores lower than 20 were discarded. To reduce bias in the analysis and artifacts, such as low-quality reads and adaptor sequences, Trimmomatic was used^93^. The trimmed reads were mapped to the reference genome *L. mexicana* reference genome (MHOMGT2001U1103) with genomic data obtained from TriTrypDB version 36 (www.tritryp.org) and to the *M. musculus* genome using the TopHat splice-aware aligner^94,95^. A maximum of two mismatches were allowed. The transcripts were assembled in Cufflinks through read alignment, providing information on the known transcripts. The expression profiles of the assembled transcripts and the abundance estimates for each sample were generated by Cufflinks^96^. The expression values were calculated as fragments per kilobase of transcript per million mapped reads (FPKM) and are represented as normalized values based on the transcript length and coverage depth^97^. Gene expression level values were calculated from the transcript counts. DEG analysis was performed for the following comparisons: (1) C57BL/6 *vs*. BALB/c, (2) BALB/c infected with *L. amazonensis vs*. BALB/c, (3) C57BL/6 infected with *L. amazonensis vs*. C57BL/6, and (4) C57BL/6 infected with *L. amazonensis vs*. BALB/c infected with *L. amazonensis*. Genes with FPKM values of zero were excluded. Groups under different conditions or with different DEGs were filtered out through statistical hypothesis tests. The false discovery rate (FDR) was controlled by adjusting the p-value using the Benjamini-Hochberg algorithm^98^. Functional annotation was performed using GO and KEGG analyses. All analyses were performed by Macrogen Inc. (Seoul, South Korea).

### RT-qPCR validation

RT-qPCR validation assays were performed using total RNA isolated as previously described above from five biological replicates. Reverse transcription was performed using 2 µg of total RNA as a template, reverse transcriptase and random primers (RevertAid H Minus Reverse Transcriptase Kit, Thermo-Scientific, Canada), according to the manufacturer’s instructions. Equal amounts of cDNA were assessed in total volumes of 25 μL containing Maxima SYBR Green qPCR Master Mix (Thermo Scientific, Lithuania, EU) and primers (200 nM) (Table S4). The mixtures were incubated at 94 °C for 5 min, followed by 40 cycles at 94 °C for 30 s, 60 °C for 30 s and 72 °C for 30 s. A negative control in the absence of reverse transcriptase was included in the RT-qPCR assays to detect DNA contamination in the RNA samples. The reactions were carried out using a PikoReal Real-time PCR System (Thermo Scientific, Finland). The reactions were performed in duplicate, and analyses were performed using PikoReal Software 2.2 (Thermo Scientific). The fold changes were calculated by relative quantification using the ΔΔCt method^99^. The data were normalized by *Gapdh* expression, and the relative gene expression was set to 1 for the control (non-infected) samples. The normalized absolute copy number of the amastin-like gene (LmxM.33.0960) was calculated based on the normalization to a reference, considering the molar mass concentration, according to a standard curve generated from a ten-fold dilution of a quantified PCR product. The normalized *Amastin*/*Gapdh* ratio of the absolute number of molecules was used as an expression parameter according to a standard curve generated from a ten-fold serial dilution of a quantified and linearized plasmid containing the target fragment.

### Statistical analysis

The experiments were performed with five biological replicates per group and the results are presented as the means ± SDs. DEGs were considered statistically significant considering fold changes ≥ 2, *p*-value < 0.05 and FDR analysis. RT-qPCR validation assays were performed with five biological replicates, and the results are presented the means ± SDs. Statistical analysis was based on Student´s *t*-test with *p*-value < 0.05 indicating statistical significance.

### Ethics statement

The experimental protocols for animals were approved by the Animal Care and Use Committee at the Institute of Bioscience of the University of São Paulo (CEUA 233/2015). This study was carried out in strict accordance with the recommended guidelines and the policies for the care and use of laboratory animals of São Paulo State (Lei Estadual 11.977, de 25/08/2005) and the Brazilian government (Lei Federal 11.794, de 08/10/2008).

## Supplementary information

Supplementary Information
