# Author Correction: Differential immune response modulation in early *Leishmania amazonensis* infection of BALB/c and C57BL/6 macrophages based on transcriptome profiles

**DOI:** 10.1038/s41598-020-61225-6

**Published:** 2020-03-04

**Authors:** Juliana Ide Aoki, Sandra Marcia Muxel, Ricardo Andrade Zampieri, Karl Erik Müller, Audun Helge Nerland, Lucile Maria Floeter-Winter

**Affiliations:** 1 Department of Physiology, Institute of Bioscience, University of São Paulo, São Paulo, Brazil; 2 Department of Clinical Science, University of Bergen, Bergen, Norway; 3 Department of Internal Medicine, Drammen Hospital, Drammen, Norway

Correction to: *Scientific Reports* 10.1038/s41598-019-56305-1, published online 27 December 2019

The Acknowledgements section in this Article is incomplete.

“We would like to thank Juliane Cristina Ribeiro Fernandes and Stephanie Maia Acuña for their comments and suggestions.”

should read:

“We would like to thank Juliane Cristina Ribeiro Fernandes and Stephanie Maia Acuña for their comments and suggestions. This work was supported by Fundação de Amparo à Pesquisa do Estado de São Paulo – FAPESP (São Paulo Research Foundation): #2016/03273-6, #2018/24693-9, #2014/50717-1, #2018/23512-0; Conselho Nacional de Desenvolvimento Científico e Tecnológico (CNPq); Coordenação de Aperfeiçoamento de Pessoal de Nível Superior (Brazilian Federal Agency for the Support and Evaluation of Graduate Education); Senter for Internasjonalisering av Utdanning (Norwegian Centre for International Cooperation in Education); Department of Biomedicine and Faculty of Medicine and Dentistry, University of Bergen.”

