# Supplementary material for: Differential immune response modulation in early Leishmania amazonensis infection of BALB/c and C57BL/6 macrophages based on transcriptome profiles
Source: Sci Rep. 2019 Dec 27;9:19841. doi: 10.1038/s41598-019-56305-1 (PMC6934472; doi:10.1038/s41598-019-56305-1)
Supplement: Supplementary file 1 — Supplementary Information [file 41598_2019_56305_MOESM1_ESM.pdf]

Differential immune response modulation in early *Leishmania amazonensis* infection of BALB/c and C57BL/6 macrophages based on transcriptome profiles

Juliana Ide Aoki, Sandra Marcia Muxel, Ricardo Andrade Zampieri, Karl Erik Müller, Audun Helge Nerland and Lucile Maria Floeter-Winter

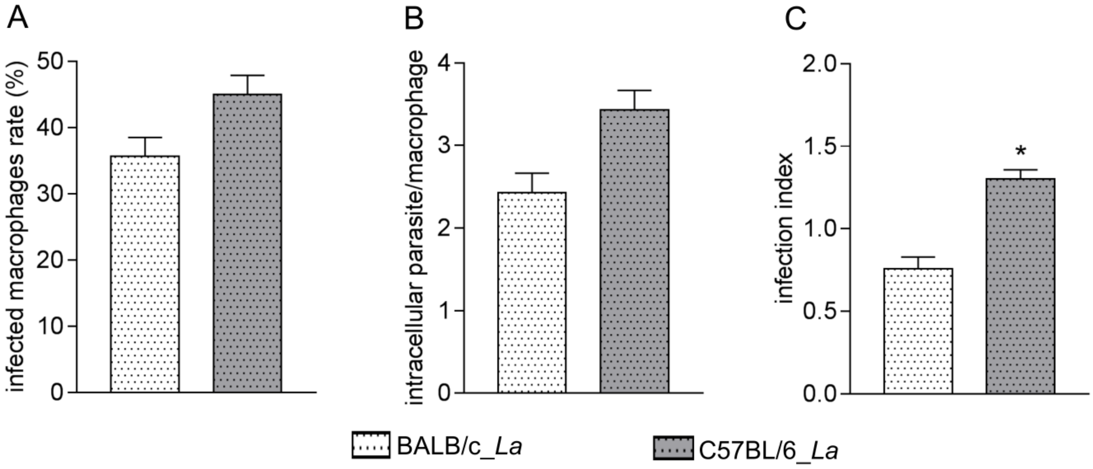

Figure S1. Infectivity evaluation of BMDMs from BALB/c and C57BL/6 infected with *L. amazonensis*. BMDMs from BALB/c and C57BL/6 mice were infected with *L. amazonensis* (MOI 5:1) and incubated at 34°C and 5% CO_2 for 4 h. **(A)** The rate of infection was determined by the percentage of infected macrophages among total macrophages. **(B)** The number of intracellular parasite per macrophage rate was determined by the number of intracellular parasites per infected macrophage. **(C)** The infection index was calculated by multiplying the rate of infected macrophages by the mean number of parasites per infected macrophage. Infections were evaluated by counting 400 panoptic-stained cells. The bars indicate the mean ± SD of three independent biological replicates. Statistical analysis was performed using the *t*-test, considering (*) *p*-value < 0.05 for the comparison of infected C57BL/6 *vs.* infected BALB/c macrophages. *L. amazonensis* (La).

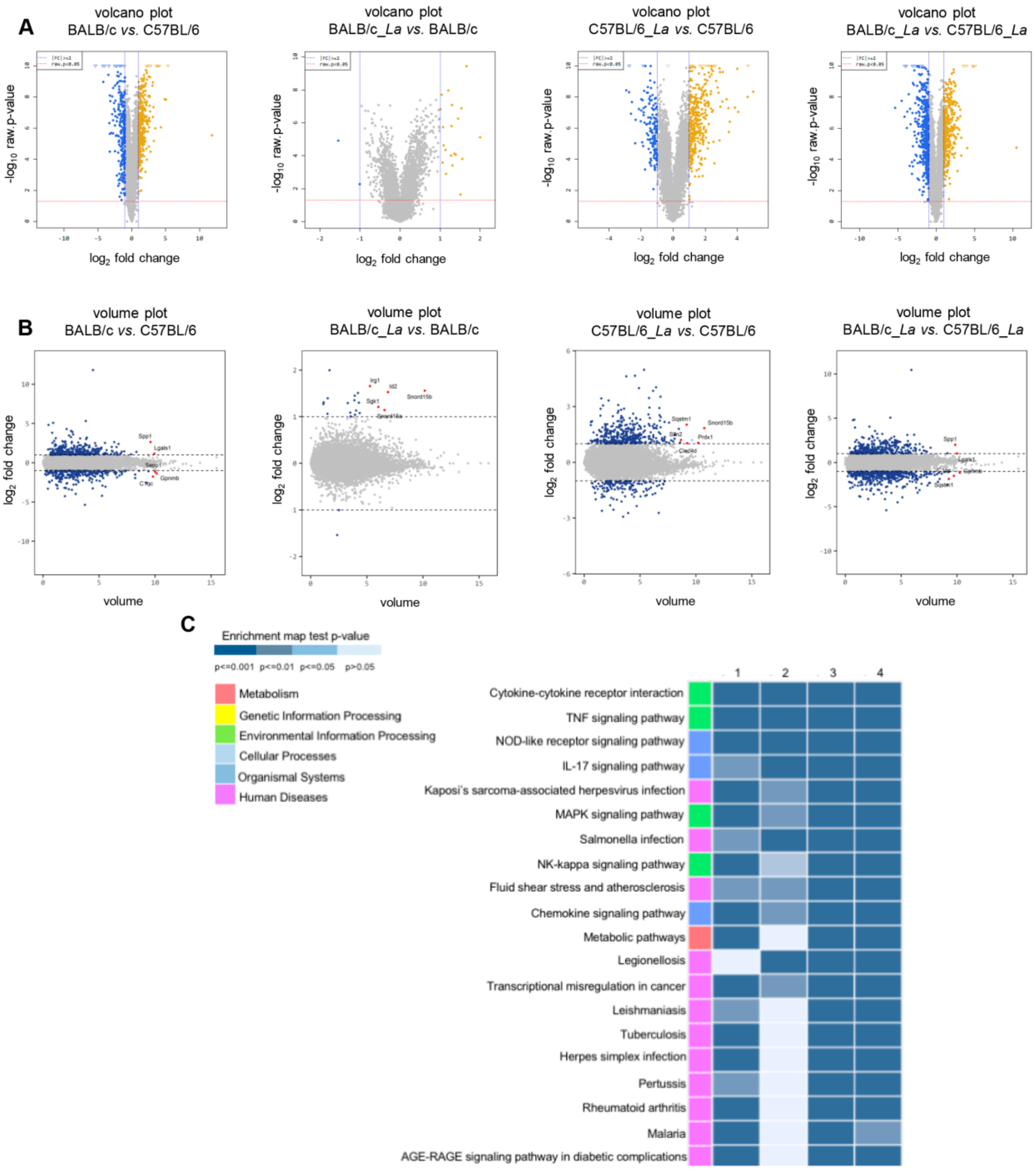

Figure S2. Transcriptome profiling of DEGs in BALB/c and C57BL/6 BMDMs infected with *L. amazonensis*. (A) Volcano plots of the comparisons, considering genes with fold change ≥ 2 and a *p*-value < 0.05, as statistically significant. Genes significantly upregulated (yellow dots) are located in the upper right square of each graph (positive log fold change value). Genes significantly downregulated (blue dots) are located in the upper left quadrant of each graph (negative log fold change value). **(B)** Volume plots of the comparisons, considering genes with $\log_2$ fold change ≥ 2 and a *p*-value < 0.05 (blue dots), as statistically significant. The five most significantly upregulated and downregulated genes are represented by red dots. **(C)** KEGG enrichment analysis showing a heat map of the 20 most regulated pathways in (1) non-infected BALB/c *vs.* non-infected C57BL/6 BMDMs, (2) BALB/c infected with *L. amazonensis vs.* non-infected BALB/c BMDMs, (3) C57BL/6 infected with *L. amazonensis vs.* BALB/c BMDMs infected with *L. amazonensis* and (4) C57BL/6 infected with *L. amazonensis vs.* non-infected C57BL/6 BMDMs. *L. amazonensis* (La).

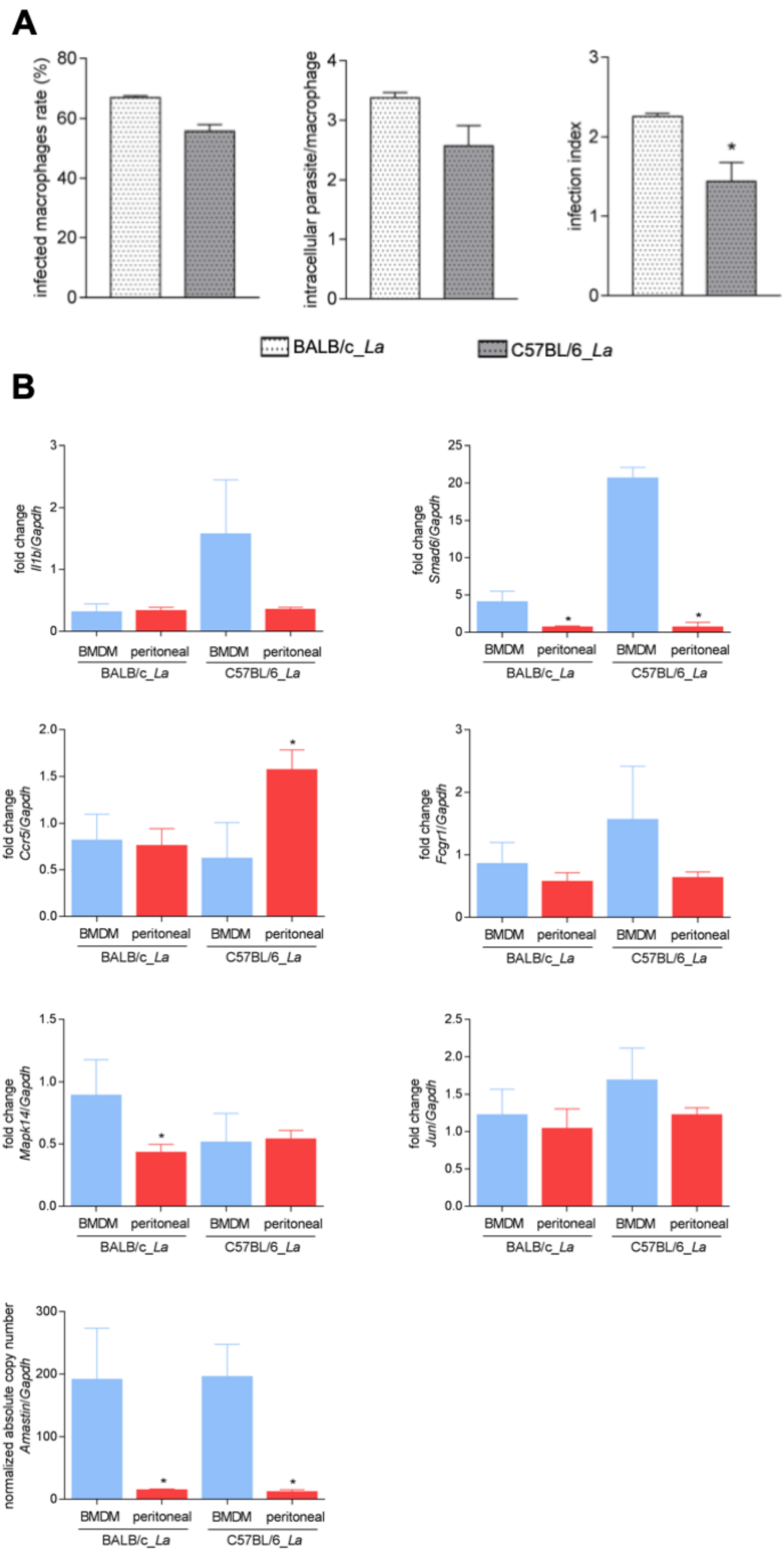

A
infected macrophages rate (%)
intracellular parasite/macrophage
infection index
BALB/c_La
C57BL/6_La
B
fold change
Il1b/Gapdh
Smad6/Gapdh
Ccr5/Gapdh
Fcgr1/Gapdh
Mapk14/Gapdh
Jun/Gapdh
normalized absolute copy number
Amastin/Gapdh
BMDM
peritoneal
BALB/c_La
C57BL/6_La

Figure S3. Infectivity evaluation of peritoneal macrophages from BALB/c and C57BL/6 infected with *L. amazonensis* and RT-qPCR of modulated genes comparing BMDMs and peritoneal macrophages. (A) Peritoneal macrophages were collected from BALB/c and C57BL/6 mice, infected with *L.amazonensis* (MOI 5:1) and incubated at 34°C and 5% CO_2 for 4 h. The rate of macrophages infection was determined by the percentage of infected macrophages among total macrophages. The intracellular parasite per macrophage rate was determined by the number of intracellular parasite per infected macrophage. The infection index was calculated by multiplying the rate of infected macrophages by the mean number of parasites per infected macrophage. Infections were evaluated by counting 400 panoptic-stained cells. The bars indicate the mean ± SD of three independent biological replicates. Statistical analysis was performed using the *t*-test, considering (*) *p*-value < 0.05 for the comparison of infected C57BL/6 *vs.* infected BALB/c macrophages. **(B)** Comparative analysis of the relative expression levels of selected genes determined by RT-qPCR. The bars represent the mean ± SD of fold change of five independent biological replicates performed in duplicate of *Il1b*, *Fcgr1*, *Ccr5*, *Smad6*, *Jun* and *Mapk14*. The fold change was calculated by the relative quantification using ΔΔCt method. The data were normalized by *Gapdh* expression and the relative gene expression was set to 1 for the control (non-infected) samples. Statistical analysis was performed using the *t*-test, considering (*) *p*-value < 0.05 for the comparison peritoneal macrophages *vs.* BMDMs in BALB/c_*La* and C57BL/6_*La* The bars of Amastin-like (LmxM.33.0960) represent the mean of normalization of *Amastin*/*Gapdh* in *La* infecting BALB/c and *La* infecting C57BL/6 macrophages. *L. amazonensis* (*La*).

Table S2. The five most differentially expressed genes of BMDMs from BALB/c and C57BL/6 in response to *L. amazonensis* infection

ID	product description	fold change	*p*-value
	BALB/c *vs.* C57BL/6		
Spp1	*secreted phosphoprotein 1*	6.26	< 0.05
Lgals1	*lectin, galactose binding, soluble 1*	2.24	< 0.05
Sepp1	*selenoprotein P, plasma, 1*	-2.21	< 0.05
Gpnmb	*glycoprotein (transmembrane) nmb*	-2.58	< 0.05
C1qc	*complement component 1, q subcomponent, C chain*	-3.38	< 0.05
	BALB/c_*La vs.* BALB/c non-infected		
Irg1	*immune responsive gene 1*	3.15	< 0.05
Id2	*inhibitor of DNA binding 2*	2.87	< 0.05
Snord15b	*small nucleolar RNA, C/D box 14B*	2.94	< 0.05
Sgk1	*serum/glucocorticoid regulated kinase 1*	2.31	< 0.05
Snord15a	*small nucleolar RNA, C/D box 15A*	2.20	< 0.05
	C57BL/6_*La vs.* C57BL/6 non-infected		
Sqstm1	*sequestosome-1 isoform 2*	4.07	< 0.05
Snord15b	*small nucleolar RNA, C/D box 14B*	3.58	< 0.05
Prdx1	*peroxiredoxin 1*	2.06	< 0.05
Slfn2	*schlafen 2*	2.30	< 0.05
Clec4d	*C-type lectin domain Family 4, member d*	2.04	< 0.05
	BALB/c_*La vs.* C57BL/6_*La*		
Spp1	*secreted phosphoprotein 1*	3.99	< 0.05
Lgals1	*lectin, galactose binding, soluble 1*	2.04	< 0.05
Gpnmb	*glycoprotein (transmembrane) nmb*	-2.23	< 0.05
C1qc	*complement component 1, q subcomponent, C chain*	-2.85	< 0.05
Sqstm1	*sequestosome-1 isoform 2*	-3.62	< 0.05

The five most highly upregulated and downregulated genes among 12,641 transcripts previously defined as DEGs in non-infected BALB/c *vs.* non-infected C57BL/6 BMDMs; BALB/c

infected with *L. amazonensis vs.* non-infected BALB/c BMDMs; C57BL/6 infected with *L. amazonensis vs.* non-infected C57BL/6 BMDMs; BALB/c infected with *L. amazonensis vs.* C57BL/6 infected with *L. amazonensis,* adjusted for fold change ≥ 2 and *p*-value < 0.05. The list is based on the volume plot of differentially expressed genes. *L. amazonensis* (*La*).

Table S3. GO enrichment analysis and DEGs profile involved in the immune system process of BMDMs from BALB/c and C57BL/6 in response to *L. amazonensis* infection

GO ID	Map Name	genes	*p*-value	FDR
2376	Immune system process	*Hist1h4m, Gbp6, Mtus1, Dusp22, Ticam1, Wwp1, Nod1, Olr1, Irak2, Npy, Glo1, H2-Q6, H2-Q9, Chaf1b, Ada, Adora2a, Adora2b, Ampd3, Ang, Birc3, Birc2, Slc7a2, Bcl2a1a, Bcl2a1d, Bcl3, Bcl6, Prdm1, Bst1, Zfp36l1, Zfp36l2, C1qa, C1qb, C1qc, Ciita, C5ar1, Casp1, Casp3, Ccnb2, Cd14, Cd24a, Cd36, Cd38, Cd4, Cd83, Cd86, Cdk6, Cdkn1a, Cdkn2b, Cebpb, Coro1a, Ccr1, Ccr3, Ccr2, Ccr5, Camp, Cnr2, Col3a1, Csf1, Ctse, Ctsh, Cx3cr1, Ednrb, Adgre1, AF251705, Colec12, Fas, Fcgr1, Fcna, Fos, Fzd5, Fzd7, Fzd8, Gbp2, Gch1, Gcnt1, Tsc22d3, Lilrb4a, Cxcl1, H2-Ab1, Cfb, H2-K1, H2-L, H2-M2, H2-DMb1, H2-Oa, H2-Q1, H2-Q2, H2-Q4, H2-Q8, H2-T22, H2-T24, Ptpn6, Hfe, Hhex, Hif1a, Hlx, Hmox1, Hsp90aa1, Hyal2, Icam1, Id2, Cxcl10, Ifit3, Cd79b, Il18bp, Il10, Il15, Il16, Il17ra, Il1a, Il1b, Il1rn, Il7r, Irf1, Irf4, Irg1, Itga4, Itgal, Jag1, Jak2, Jun, Junb, Kdr, Klf4, Mafb, Lcn2, Lcp2, Lfng, Lgals1, Lmo4, Blnk, Tlr8, Ly86, Lyl1, Smad3, Smad6, Mapkapk2, Marco, Mef2c, Mertk, Cxcl9, Mitf, Mmp14, Mmp9, Clec4d, Cited2, Mx1, Myc, Nck1, Ndrg1, Nfatc2, Nfe2l2, Nfkb1, Nfkb2, Nfkbia, Notch1, Notch2, Slc11a1, Slc11a2, Sqstm1, Osm, Bloc1s6, Prdx1, Pde4b, Pdgfrb, Pik3cd, Serpine1, Pnp, Procr, Ptafr, Ptger4, Ptprc, Ripk2, Ptprj, Ptpro, Rbpj, Rela, Relb, S100a8, Ccl2, Ccl3, Ccl4, Ccl5, Ccl7, Ccl9, Cxcl2, Skil, Slfn1, Slpi, Sod2, Spn, Spp1, Src, Samd9l, Syk, Trib1, Tapbpl, Tacc3, Mb21d1, Il1f9, Rassf2, Nlrp3, Cd300a, Tgfbr1, Tgtp1, Thbs1, Tlr1, Tnf, Tnfaip3, Tnfrsf1b, Cd40, Tnfsf8, Tnfsf9, Top2a, Traf3, Traf6, Tfrc, Nr1h3, Vav1, Vcam1, Vegfa, Ezr, Nrros, Treml4, Ticam2, Rab7b, Zfp36, Gbp5, Gbp7, Themis2, Oasl1, Ppp4r2, Cd109, Zc3h12d, Gpr68, Clec5a, Fyb, Gadd45g, Sh2b2, Oasl2, Malt1, Tlr2, Mill2, Nfkbid, Chd2, Tnfrsf26, Fcgr4, Cd300lf, Il27, Nod2, Axl, Map3k8, Mapk14, Clec4a2, Nlrc4, Ahcy, Pla2g7, Nbn, Vsig4, Pik3cg, Hist1h3d, Hist1h3b, Hist1h3e, Hist1h3h, Hist1h3i, Hist2h3b, Hist1h4c, Hist1h4d, Hist1h4f, Hist1h4i, Hist1h4j, Hist1h4k, Hist1h4n, Hist1h2ba, Hist1h2be, Hist1h2bf, Hist1h2bg, Hist1h2bk, Hist1h2bl, C5ar2, Hist4h4, Gpr183, Hist1h4a, Hist1h4b, Treml2, Cxcl3, Hist1h3a, Batf3, Itgad, Aim2, Irs2, Tnip3, Icosl, Tnfsf14, Batf, Slc40a1, Irf7, Irgm2, Mefv, Gbp3, Samhd1, Htra1, Ccl24, Tbk1, Foxo3, Ripk3, Clec4e, Clec4n, Rabgef1, Lat2, Clec1b, Stap1, Cxcl14, Tnip1, Pmaip1, Rrs1, Erbb2ip, Iigp1, Cd274, Dusp10, Asb2, Tmem176b, Tmem176a, Ifitm3, Rgcc, Trim13, Ifit3b, Ifit1bl1, Gm8909, Atg12, Samsn1, Rab32, Hist1h2bc, Ifitm1, Hist1h4h, Bst2, Hilpda, Tnfsf13, Tnfaip8l2, Alpk1, Snx10, Irak3, Sash3, Batf2, Stx11, Trim14, Ddit4, Rnf19b, Slamf7, Armc6, Apobec3, Fam20c, Hcar2, Clec2i, Clec2d, Trim34a, Hist2h3c2, Hist2h4, Hist1h3g, Phlpp1, Tiparp*	$1.63e^{-164}$	$5.82e^{-162}$

Gene Ontology (GO) enrichment analysis and profile of differentially expressed genes (DEGs) in BALB/c and C57BL/6 BMDMs in response to *L. amazonensis* infection, highlighting the immune system process (Map name). Analysis was based on p-values and false discovery rates (FDR).

Table S4. List of primers used for RT-qPCR validation for *M. musculus* and *L. amazonensis*

M. musculus primers	5´- 3´
*Gapdh*_F	GGCAAATTCAACGGCACAGT
*Gapdh*_R	CCTTTTGGCTCCACCCTTCA
*Il1b*_F	CCAAGCTTCCTTGTGCAAGTG
*Il1b*_R	CTGTCAAAAGGTGGCATTTCAC
*Smad6*_F	TCTGCGGGCCAGAATCAC
*Smad6*_R	ACAATGTAGAATCGGACAGATCCA
*Fcgr1*_F	TTGGAGATGACATGTGGCTTCT
*Fcgr1*_R	AGCCTTGGTGGCATTAACCA
*Ccr5*_F	CTAGCCAGAGGAGGTGAGACATC
*Ccr5*_R	GGAACTGACCCTTGAAAATCCA
*Jun*_F	CATCCGTTTGTCTTCATTTTCTCA
*Jun*_R	CCAAATGCTCCCCAAAATACC
*Mapk14*_F	CGAGAGTTGCGTCTGCTGAAG
*Mapk14*_R	AGTGACCTTGCGGGTGTGAAC
***L. amazonensis* primers**	**5´- 3´**
*Gapdh*_F	TCAAGGTCGGTATCAACGGC
*Gapdh*_R	TGCACCGTGTCGTACTTCAT
Amastin-like_F	GGAGCGCTACTTCAGCTATGGA
Amastin-like_R	CGGATCATCAATAAGACGATGTTG

Oligonucleotides sequences used in RT-qPCR validation assays. *Glyceraldehyde 3-phosphate dehydrogenase* (*Gapdh*). *Interleukin* β (*Il1b*). *Mothers against decapentaplegic homolog 6* (*Smad6*). *High affinity immunoglobulin gamma Fc receptor I* (*Fcgr1*). *C-C chemokine receptor type 5* (*Ccr5*). *Transcription factor AP-1* (*Jun*). *Mitogen-activated protein kinase 14* (*Mapk14*). *Amastin*-like (LmxM.33.0960). Forward (F) and Reverse (R).
